# First Family of *MATR3*-Related Distal Myopathy From Italy: The Role of Muscle Biopsy in the Diagnosis and Characterization of a Still Poorly Understood Disease

**DOI:** 10.3389/fneur.2021.715386

**Published:** 2021-10-01

**Authors:** Michele Cavalli, Rosanna Cardani, Laura Valentina Renna, Mauro Toffetti, Luisa Villa, Giovanni Meola

**Affiliations:** ^1^Université Côte d'Azur, Peripheral Nervous System and Muscle Department, Pasteur 2 Hospital, Centre Hospitalier Universitaire de Nice, Nice, France; ^2^BioCor Biobank, Department of Clinical Pathology, Istituto di Ricovero e Cura a Carattere Scientifico - IRCCS Policlinico San Donato, San Donato Milanese, Italy; ^3^Department of Neurology and Stroke Unit, ASST Franciacorta, Chiari, Italy; ^4^Department of Biomedical Sciences for Health, Department of Neurorehabilitation Sciences, Casa di Cura del Policlinico, University of Milan, Milan, Italy

**Keywords:** MATR3, muscular biopsy, multisystem proteinopathy, distal myopathy with vocal cord paralysis, motor neuron

## Abstract

Mutations in the *MATR3* gene are associated to distal myopathy with vocal cord and pharyngeal weakness (VCPDM), as well as familiar and sporadic motor neuron disease. To date, 12 VCPDM families from the United States, Germany, Japan, Bulgary, and France have been described in the literature. Here we report an Italian family with a propositus of a 40-year-old woman presenting progressive bilateral foot drop, rhinolalia, and distal muscular atrophy, without clinical signs of motor neuron affection. Her father, deceased some years before, presented a similar distal myopathy phenotype, while her 20-year-old son is asymptomatic. Myopathic changes with vacuolization were observed in muscle biopsy from the propositus. These results, together with the peculiar clinical picture, lead to *MATR3* gene sequencing, which revealed a heterozygous p.S85C mutation in the propositus. The same mutation was found in her son. Over a 5-year follow-up, progression is mild in the propositus, while her son remains asymptomatic. Clinical, radiological, and pathological data of our propositus are presented and compared to previously reported cases of VCPDM. VCPDM turns out to be a quite homogenous phenotype of late-onset myopathy associated to p.S85C mutation in *MATR3* gene. *MATR3*-related pathology, encompassing myopathy and motor neuron disease, represents an illustrative example of multisystem proteinopathy (MSP), such as other diseases associated to mutations in *VCP, HNRNPA2B1, HNRNPA1*, and *SQSTM1* genes. The present report contributes to a further characterization of this still poorly understood pathology and points out the diagnostic utility of muscle biopsy in challenging cases.

## Introduction

Distal myopathies are a heterogeneous group of hereditary skeletal muscle diseases characterized by distal muscular wasting at onset. Due to their rarity and a still ill-defined nosology, diagnosis is challenging.

Differential diagnosis is wide, including myotonic dystrophy type 1 (DM1), facioscapulohumeral muscular dystrophy (FSHD), inclusion body myositis, motor neuron disease, Charcot-Marie-Tooth disease (CMT), and acquired motor neuropathies. Moreover, various abnormalities in many different molecular pathways could lead to a distal myopathy phenotype. Disruption of muscular fiber structure, myofibrillar disarray, or glycogen or protein cytoplasmic accumulation is associated to distal myopathy (1).

In most cases, muscle biopsy shows non-specific dystrophic features with vacuolization when standard stains are used. Nonetheless, further clinical, anatomopathological, and radiological characterization of these pathologies can be relevant in orienting proper genetic analysis (2).

Since 2009, p.S85C mutation in *MATR3* gene is known to be causative of an autosomal dominant form of distal myopathy with vocal cord and pharyngeal weakness (VCPDM) (3). Since then, 12 families from the United States (3–5), Bulgary (3), Germany (6), Japan (7), and France (8) have been reported for presenting a distal myopathy phenotype in association with this specific mutation.

Here we report the first Italian family presenting VCPDM, the propositus being a 40-year-old woman consulting for progressive bilateral foot drop. Muscular weakness in ankle dorsiflexion appeared 9 months earlier. At clinical examination, rhinolalia and distal muscular atrophy of the four limbs were observed. Remarkably, atrophy of the first dorsal interosseus and thenar muscles was outstanding, while hypothenar eminence was relatively spared, resulting in a split-hand feature. Muscular weakness was mild in first and second finger abductors (4 MRC scale), moderate in neck flexors (3 MRC scale), and severe in ankle dorsiflexors (2 MRC scale). Osteotendinous reflexes were normal and no sensory defect was detected. No fasciculation or any sign of upper motor neuron affection was clinically evident. Deglutition was unaffected. Vital capacity was preserved, being 98% of the predicted, with no significant difference between ortho- and clinostatism. Twelve-lead ECG, transthoracic echocardiogram, and 24-h ECG monitoring were unremarkable. Serum CK was mildly elevated up to 189 U/L.

Her father had been deceased in his early fifties due to a cardiac event, some years before the onset of the disease in the propositus. Nonetheless, his medical records report of a progressive distal muscular wasting in the four limbs since his late thirties.

The 20-year-old son of the propositus was asymptomatic and did not show any abnormality at the clinical examination.

Over a 5-year follow-up, progression in the propositus is mild, so that deglutition, respiration, and ambulation are preserved. Nonetheless, muscular weakness is more evident in the first and second finger abductors (3 MRC scale) and extended to the posterior legs, notably affecting ankle plantar flexors (3 MRC scale). Her son is still asymptomatic.

## Methods

### Patients and Skeletal Muscle Samples

This study was authorized by the Institutional Ethics Committee (ASL MI2-Melegnano via VIII Giugno, Milan) and was conducted according to the principles expressed in the Declaration of Helsinki, the institutional regulation, and Italian laws and guidelines. All blood samples and muscle biopsies were used for this study after receiving written informed consent from the patients. Muscle biopsies were taken under sterile conditions from the propositus (vastus lateralis), one patient affected by myotonic dystrophy type 1 (tibialis anterior), and one healthy patient (biceps brachii) used as control.

### Muscle Histopathology and Immunostaining

Muscle tissue was fresh-frozen in isopentane cooled in liquid nitrogen. Histopathological analysis was performed on serial sections (8 μm) processed for routine histological or histochemical staining. Serial transverse muscle cryostat sections 6 μm were incubated with 3% hydrogen peroxide. Non-specific binding sites were blocked with normal goat serum (NGS; DAKO) at a dilution of 1:20 in PBS containing 2% bovine serum albumin (BSA; Sigma–Aldrich, St. Louis, MO, USA) for 20 min at room temperature (RT). Unfixed sections were incubated with mouse monoclonal primary antibodies against four different myosin heavy chain (MHC) isotypes: MHCfast (1:400 in PBS + 2% BSA for 1 h at RT; Sigma–Aldrich), MHCslow (1:400 in PBS + 2% BSA for 1 h at RT; Sigma–Aldrich), MHC-developmental (1:20 in PBS + 2% BSA overnight at 4°C; Novocastra), MHC-neonatal (1:10 in PBS + 2% BSA for 1 h at RT; Novocastra). Paraformaldehyde fixed sections (4% in PBS for 15 min at 4°C) were incubated with rabbit polyclonal antibody anti TDP-43 (1:400 in PBS + 2% BSA overnight at 4°C; Proteintech). For SMI-31 immunostaining, acetone fixed sections were incubated overnight at 4°C with rabbit polyclonal antibody at 1:2000 in PBS + 2% BSA (Novocastra). After washing in PBS, sections were incubated with goat anti-mouse or goat anti-rabbit biotinylated secondary antibody diluted 1:300 in PBS + 2% BSA for 1 h at RT. Sections were incubated with StreptABComplex (DAKO) for 30 min and then exposed to the 3,3′-diaminobenzidine tetrahydrochloride (DAB) chromogen reaction solution for 10 min. Nuclei were counterstained with Mayer's hematoxylin. For matrin-3 immunofluorescence, sections were fixed with paraformaldehyde (4% in PBS for 15 min at 4°C) and then incubated with a rabbit polyclonal primary antibody applied overnight at 4°C at a dilution of 1:500 in PBS + 2% BSA (Novus Biologicals, Littleton, CO, USA). After incubation with a goat anti-rabbit Alexa488-labeled antibody (1:200 in PBS + 2% BSA, 1 h at RT; Molecular Probes), nuclei were stained with DAPI (1 lg/ml; Sigma–Aldrich, St. Louis, MO, USA), and sections were mounted with Mowiol (Calbiochem, Milan, Italy). As controls, some slides were processed as described above but omitting the incubation with the primary antibody.

## Results

Electromyography (EMG) in our propositus revealed fibrillations, small motor unit action potentials (MUAP), and myopathic recruitment in distal muscles of the four limbs.

In her father's medical records, there is mention of EMG features consistent with irritable myopathy.

**Figure 1:**
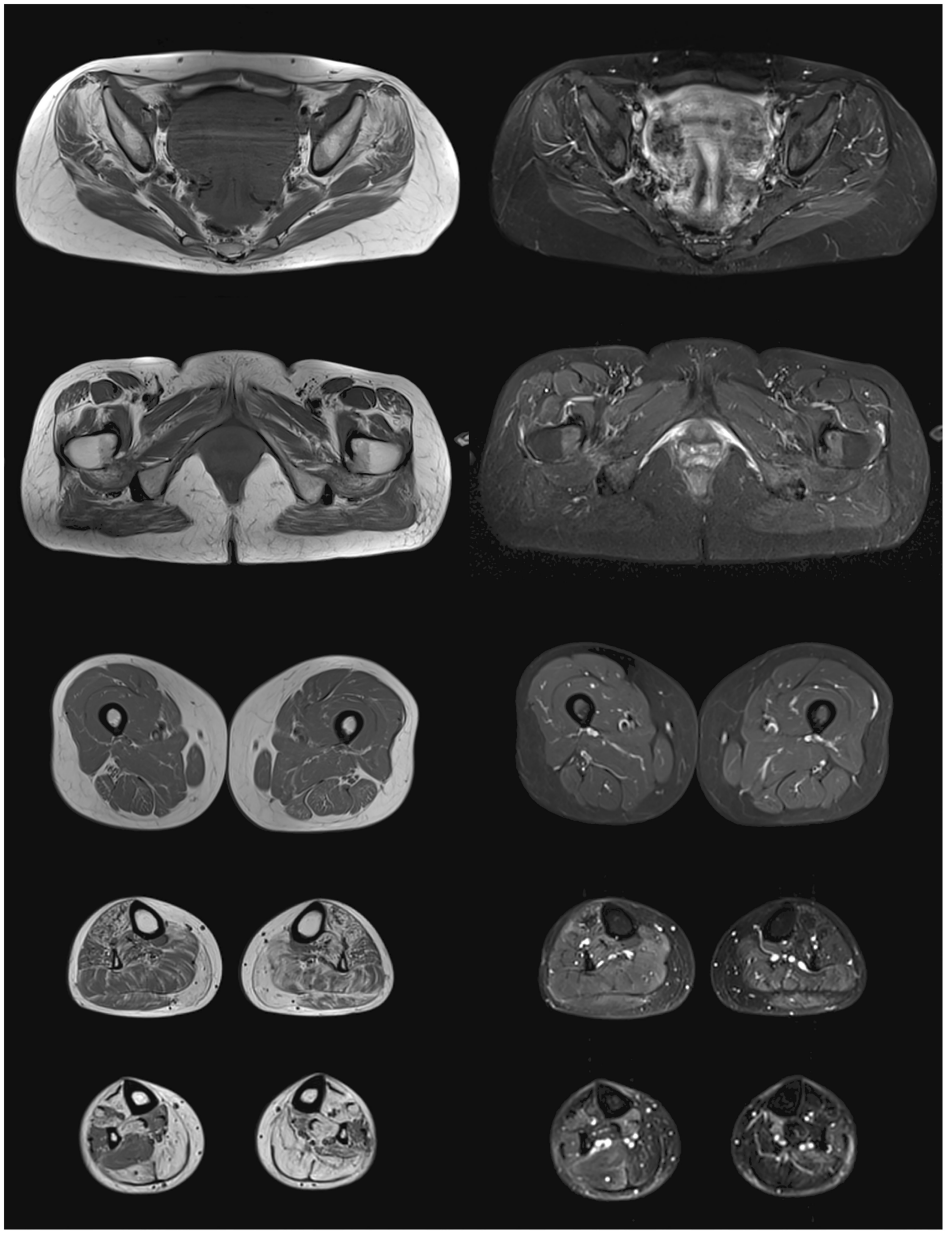
Lower body skeletal muscle MRI from the propositus. On the left panel, T1 images showing fatty degeneration as hyperintense signal in *gluteus minimus* and *medius, adductor maximus, quadratus femoris, tibialis anterior, gastrocnemius*, and *soleus*. On the right panel, STIR images showing interstitial edema as hyperintense signal in partially infiltrated muscles of the legs.

Lower-body skeletal muscle MRI of our propositus found bilateral fatty degeneration mainly in the *gluteus minimus* and *medius, adductor maximus, quadratus femoris, tibialis anterior, gastrocnemius*, and *soleus*. All leg muscles spared from total fatty infiltration showed some STIR bright signal, usually associated to interstitial edema (Figure 1).

No muscular imaging is available from her father, and her son refused to undergo MRI.

**Figure 2:**
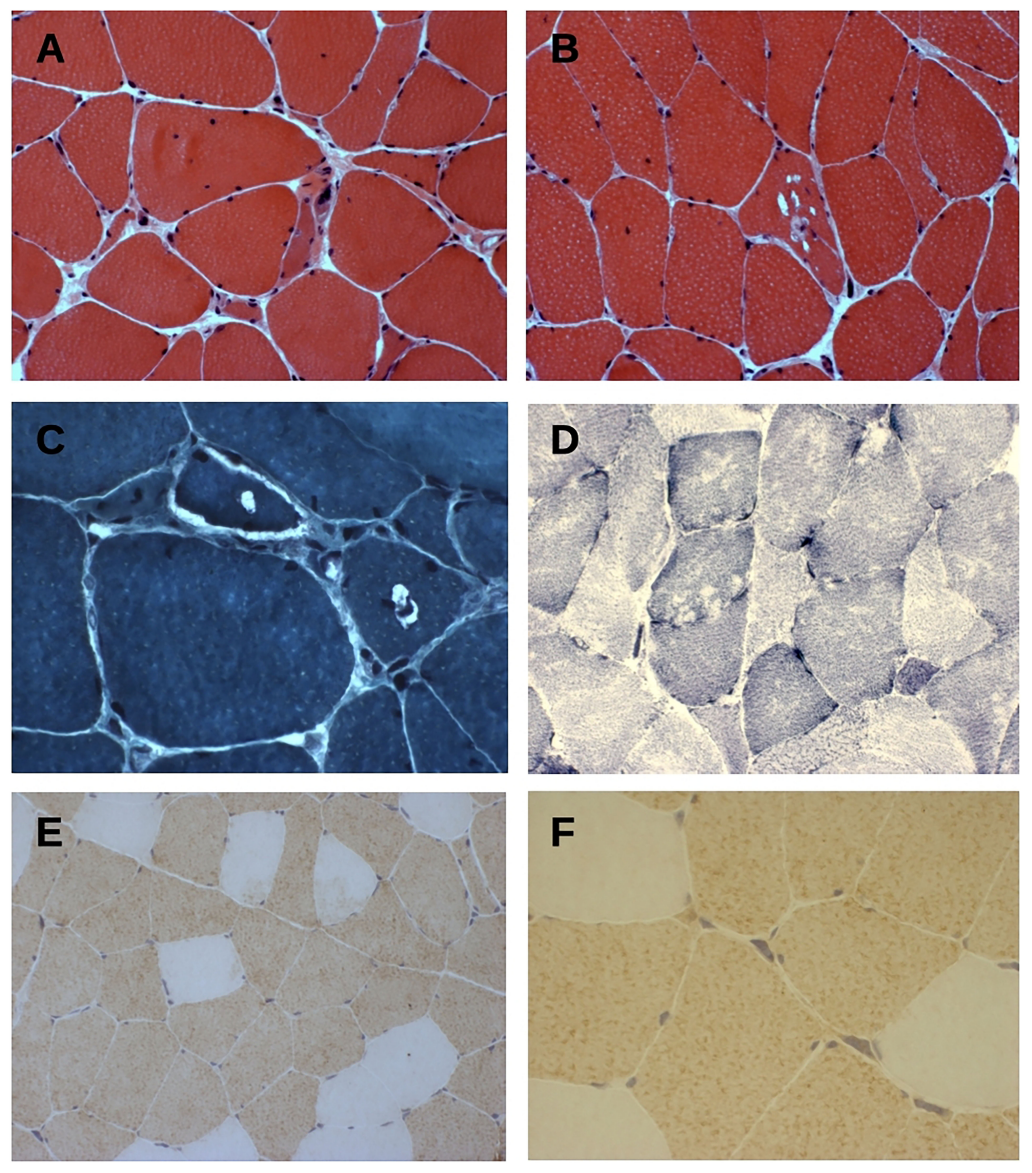
**(A–C)** Hematoxylin & eosin **(A,B)** and trichrome Gomori **(C)** staining of tranverse muscle sections shows fiber size variability, nuclear centralization, and fibers with rimmed-vacuolar changes. Original magnification 200× **(A,B)** and 400× **(C)**. **(D)** NADH-TR staining show vacuoles mainly in type 1 fibers. Original magnification 200×. **(E,F)** Fast myosin heavy chain (MyHCfast) immunostaining shows a type 2 fiber predominance, a variation in fiber size, and some MyHCfast-positive nuclear clumps. Original magnification 200× **(E)** and 400× **(F)**.

Histopathological findings on muscle biopsy showed increased fiber size variability, mild endomysial fibrosis, rare nuclear internalization, nuclear clumps and some rimmed vacuoles, mainly in atrophic fibers (Figures 2A–C). Immunochemistry revealed type 2 fiber (MHCfast positive fibers) predominance, without overt type grouping (Figures 2D–F). No significant modification was observed in oxidative or acid phosphatase reactions. Immunostaining for neonatal MHC, developmental MHC, SMI-31, and TDP-43 was negative.

**Figure 3:**
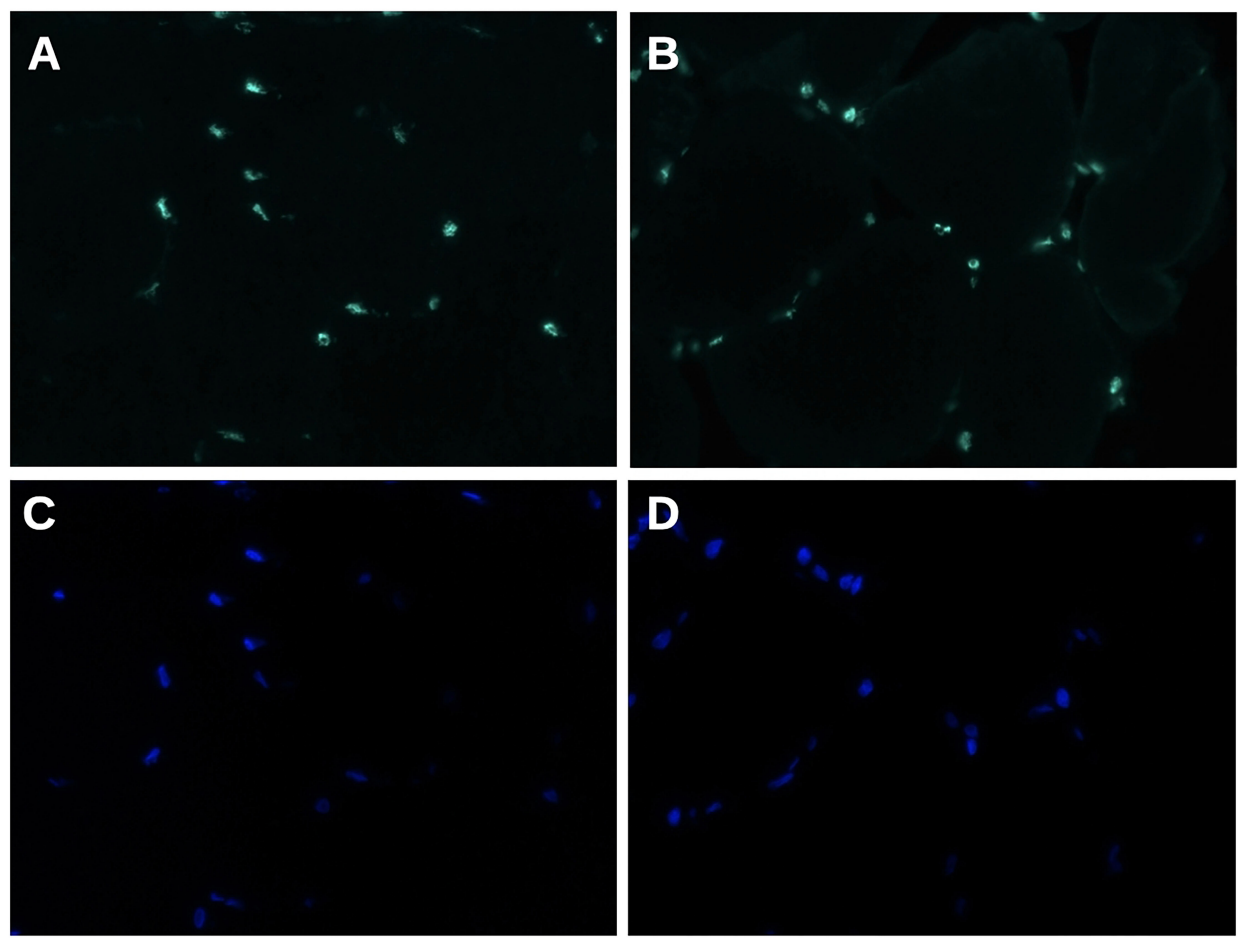
Matrin 3 immunofluorescence staining **(A,B)** shows a uniform nuclear reactivity both in normal control **(A)** and in propositus muscle **(B)**. **(C,D)** Nuclei stained with DAPI. Original magnification 400×.

Immunofluorescence analysis did not find any significant difference in matrin-3 protein expression and nuclear localization between our propositus and healthy control (Figure 3).

Clinical picture and muscle biopsy results lead to target *MATR3* gene for Sanger sequencing in our propositus, revealing a heterozygous p.S85C mutation. After adequate genetic counseling, her asymptomatic son accepted to undergo *MATR3* gene sequencing, which found the same mutation.

## Discussion

Autosomal dominant late-onset distal myopathy associated with pharyngeal weakness (VCPDM) was described and then associated to p.S85C mutation in *MATR3* gene during the first decade of the 21st century by Feit et al. (3, 4, 9).

The comparison of clinical data from our propositus to previously reported VCPDM cases points out a quite homogenous picture (see Supplementary Material for details).

The age at onset ranges from 30 to 63 years. Distal muscular weakness is the more represented symptom at onset, notably at lower limbs, reaching a figure of 78% of cases, while the peculiar bulbar palsy is reported in only 11% of cases at earlier stages. Throughout the course of the disease, some bulbar palsy became evident in 58% of cases, while distal muscular weakness is almost constant between VCPDM patients. Some degree of proximal muscular weakness could appear in later stages, being observed in almost half of the cases. However, ambulation is rarely compromised, with more than 90% of patients remaining ambulatory. Axial weakness and myalgias are rarer but possibly underreported. Respiratory function is altered in more than one-third of reported cases. Few cases presenting cardiopathy are observed, but there is no clear correlation with VCPDM. CK elevation can be observed in almost 70% of cases but it is usually mild.

Few years after the first association of VCPDM to *MATR3* gene, several mutations in the same gene, including p.S85C, were found to be causative of familial amyotrophic lateral sclerosis (ALS). This discovery led the same authors of the first VCPDM report to reconsider the initial diagnosis of myopathy, proposing instead a sort of slowly progressing motor neuron disease (10). Since then, the reports of familial ALS linked to *MATR3* gene from all over the world grew up considerably (11–14), and matrin-3 is found to be a component of cytoplasmic inclusions of motor neurons even in sporadic ALS (15). Nonetheless, the existence of a truly myopathic phenotype due to p.S85C mutation in *MATR3* gene is corroborated by several papers (5–8), as by our family.

Notably, some clinical signs commonly associated to ALS, such as brisk reflexes or split-hands, are reported in few myopathic cases (5, 10) and are observed in our propositus too.

In the present family and in reported cases of VCPDM, EMG findings are consistent with myopathy with signs of sarcolemmal electrical instability in most of patients. However, some neuropathic features are observed in more than a half of VCPDM cases. A small prospective study focusing on needle electromyography findings in eight patients from four VCPDM German families does not find any fasciculation, neither neurogenic modifications of MUAP, while features of irritable myopathy are prevalent (16).

Skeletal muscle MRI reveals fatty degeneration in distal muscles of lower limbs in more than 80% of reported cases, but proximal muscles are not spared since hamstrings and paraspinal muscles are affected in 75 and 69% of patients, respectively.

A recent study from Mensch et al. analyses whole-body muscle MRI findings from 15 VCPDM German patients. It is noteworthy that predominant posterior leg involvement in earlier stages of diseases seems to contrast to bilateral foot drop presentation (17). In our propositus, as in most of previously reported cases, this discrepancy is not evident, probably because of later stages of disease at the time of MRI realization. Even more interestingly, the cited work proposes a specific pattern of muscular involvement in VCPDM, which includes distal leg but also proximal muscles as thoracic paraspinal musculature, deltoids, and glutei (17). According to Mensch et al., only whole-body muscle MRI yields specificity when compared to other distal myopathies, but in our propositus, as in most of previously reported cases, lower-body MRI was implemented, which represents a limitation in available data.

Our propositus shows clear STIR bright signal in most of his leg muscles, and a similar finding is described in two patients from the German series reported by Müller et al. (6). The real prevalence of such modifications cannot be established, since other works do not mention STIR or T2-weighted sequences. This finding is considered a marker of muscle inflammation, while no inflammatory change is evident in muscle biopsy from our propositus, nor from previously reported cases. A possible explanation for this discrepancy could be the limited distribution of STIR hyperintensity to distal muscles, since biopsy is rarely performed on leg muscle in clinical practice.

With regard to muscle biopsy data, myopathic changes are largely predominant, being reported in 82% of biopsies. Rimmed vacuoles are observed in 59% of them. On the other hand, type grouping and other neurogenic changes are infrequent, being found in 14% of biopsies.

Notably, type 2 fiber predominance, commonly associated to neurogenic changes, is observed in our propositus and in one patient from the study of Yamashita et al. (7), possibly implicating some motor neuron involvement. Moreover, given the absence of clear fiber splitting in our propositus, some degree of neurogenic atrophy could be implicated, supporting this hypothesis.

Palmio et al. describe a significant increase in developmental MHC, while staining for neonatal MHC, a marker of neurogenic atrophy, is marginal (5). These findings are not reproduced in our propositus, in whom no abnormal MHC expression is observed.

The same authors document TDP-43 and SMI-31 accumulation in rimmed vacuoles, while staining for these proteins is negative in our sample. Similar to other myopathies with rimmed vacuoles, various proteins can form aggregates, as an end-stage phenomenon, possibly explaining discrepancy in specific stains.

Coherent with previously reported mouse models and human findings (18, 19), mutated matrin-3 is not mislocated out of the nucleus in VCPDM.

Moreover, Palmio et al. found a reduction in matrin-3 expression in the nuclei, while this observation is not confirmed by a systematic work by Mensch et al. (18). Taken together, these data are against a loss-of-function mechanism in VCPDM.

Matrin-3 is a nuclear matrix DNA/RNA-binding protein implicated in splicing regulation, mainly interacting with TDP-43 (20, 21). Notably, it is proven to play a critical role in myogenesis, interacting with PABN1 and binding various coding and non-coding myogenic transcripts (22).

Both ALS- and VCPDM-associated mutations seem to interfere with mRNA nuclear export, leading to mRNA sequestration in the nucleus (23), while they do not impair the ability of matrin-3 to interact with its physiological binders (24). Remarkably, *Drosophila* muscle seems to be specifically susceptible to the deleterious effect of p.S85C mutation in *MATR3* gene, compared to ALS-linked p.F115C mutation (25).

In analogy to Welander distal myopathy, impairment of stress granule formation is proposed as a possible pathogenic mechanism for VCPDM, since cytoplasmic granules containing stress granule components, TIA1 cytotoxic granule-associated RNA binding protein (TIA1) and G3BP stress granule assembly factor 1 (G3BP1), are observed in muscular samples by Palmio et al. (5), and p.S85C-mutated matrin-3 reduces cellular stress response *in vitro* (18).

Of note, a *MATR3*-mutated mouse model bearing p.S85C presents dysregulated protein degradation and nuclear function not only in muscular tissue but also in spinal cord motor neurons (26).

The term multisystem proteinopathy (MSP) was introduced to define a group of hereditary diseases characterized by deleterious protein accumulation in brain and spinal cord neurons, skeletal muscle, and other organs (27). Initially encompassing the inclusion body myopathy, Paget disease, frontotemporal dementia (IBMPFD) motor neuron disease spectrum, which includes *VCP, HNRNPA2B1*, and *HNRNPA1* genes (MSP1, MSP2, and MSP3, respectively), MSP classification now incorporates the pathologies associated to *SQSTM1* (MSP4), *MATR3* (MSP5), *TIA1*, and eventually *OPTN* genes (28).

## Conclusion

Due to its rarity and its complex molecular background, VCPDM remains a still poorly understood clinical entity. As in our propositus case, muscular biopsy is an irreplaceable diagnostic tool in such challenging cases.

Furthermore, our results contribute to delineate a specific myopathic phenotype in the context of *MATR3*-related disease spectrum, which encompass muscular and motor neuron pathology.

Even though clinical, pathological, and molecular characterization of *MATR3*-related disease is still far from exhaustive and many contradictory findings remain mostly unexplained, a growing amount of evidences point out the outstanding role of muscle pathology in clinical and scientific comprehension of MSP.

## Data Availability Statement

The original contributions presented in the study are included in the article/Supplementary Material, further inquiries can be directed to the corresponding author.

## Ethics Statement

The studies involving human participants were reviewed and approved by the Institutional Ethics Committee (ASL MI2-Melegnano via VIII Giugno, Milan). The patients/participants provided written informed consent to participate in the study.

## Author Contributions

MC: clinical evaluation, review of the literature, writing, and editing of the manuscript. RC: histopathology and immunostaining of muscle biopsy, editing of the images, revision, and editing of the manuscript. LVR: histopathology and immunostaining of muscle biopsy, and editing of the images. MT: clinical evaluation, review of the literature, and revision of the manuscript. LV: muscle biopsy and imaging interpretation, review of the literature, and revision of the manuscript. GM: clinical evaluation, muscle biopsy interpretation, and revision of the manuscript. All authors contributed to the article and approved the submitted version.

## Funding

GM was supported by a grant from Fondazione Malattie Miotoniche (FMM), Milan, Italy.

## Conflict of Interest

The authors declare that the research was conducted in the absence of any commercial or financial relationships that could be construed as a potential conflict of interest.

## Publisher's Note

All claims expressed in this article are solely those of the authors and do not necessarily represent those of their affiliated organizations, or those of the publisher, the editors and the reviewers. Any product that may be evaluated in this article, or claim that may be made by its manufacturer, is not guaranteed or endorsed by the publisher.

## Supplementary Material

Supplementary Material Clinical and paraclinical data from reported cases of VCPDM in the literature. LL, lower limbs; UL, upper limbs; VC, vital capacity; TA, tibialis anterior; CRD, complex repetitive discharges; RV, rimmed vacuoles.
